# Buffalo Medical Journal

**Published:** 1846-07

**Authors:** 


BUFFALO MEDICAL JOURNAL.

This journal has entered upon its second year in not only an enlarged size but a greatly improved dress, a prosperity creditable to its subscribers and industrious editor, and most gratifying to all who take an interest in the medical literature of our country. Among a host of able editors, we know not where we should go to find one more independent, impartial, and zealous than the conductor of the Buffalo Medical Journal and Monthly Review. Dr. Flint holds a ready, pointed, and vigorous pen, and the number of his Journal before us shows how industriously he is using it for the edification of his readers. He is worthy of patronage, and we hope will receive it in large measure.

